# Phytochemical Screening, Antioxidant Effect and Sperm Quality of the *Bomba ceiba* Stamen Extracts on Charolais Cattle Sperm Induced by Ferrous Sulfate

**DOI:** 10.3390/plants13070960

**Published:** 2024-03-26

**Authors:** Jiraporn Laoung-on, Sakaewan Ounjaijean, Paiwan Sudwan, Kongsak Boonyapranai

**Affiliations:** 1 Office of Research Administration, Chiang Mai University, Chiang Mai 50200, Thailand; jiraporn.l@cmu.ac.th; 2 Research Institute for Health Sciences (RIHES), Chiang Mai University, Chiang Mai 50200, Thailand; sakaewan.o@cmu.ac.th; 3 Department of Anatomy, Faculty of Medicine, Chiang Mai University, Chiang Mai 50200, Thailand; paiwan.sudwan@cmu.ac.th

**Keywords:** phytochemicals, cotton tree, bioactive compounds, natural antioxidant, sperm quality, sperm function, male reproductive system, health benefits

## Abstract

Orange *Bombax ceiba* (*B. ceiba*) is an indigenous plant, and its stamen is an important ingredient in traditional Lanna food. There are limitations in scientific reports on the effects of the biological activities of *B. ceiba* stamens on the male reproductive system. This study aims to investigate the phytochemical compounds of the orange *B. ceiba* stamen and its potential effect on the antioxidant properties and quality of cattle sperm treated with Fe. The orange BUE had the highest total phenolics, total tannins, total monomeric anthocyanins, and maximal antioxidant potential. The orange BAE had the highest concentration of total flavonoids. LC-QTOF/MS showed that the orange BUE contained the highest number of phytochemical compounds related to male reproductive enhancement. The orange BUE enhanced sperm motility, and both the orange BUE and the BAE enhanced sperm viability and normal sperm morphology via free radical scavenging. It might be suggested that *B. ceiba* stamens have benefits for sperm preservation, sperm quality, and increasing the economic value of local plants, and that they may be developed and used to guard against oxidative stress from cryodamage induced by frozen semen technology.

## 1. Introduction

*Bombax ceiba* L. (*B. ceiba*), which belongs to the Bombacaceae family [1], is commonly called the kapok tree, silk cotton tree, and Indian bombax; it is also known as “Ngui” in Thailand. It is a spectacular flowering tree with a height of up to 40 m [2]. This plant is widely found and grown in Africa, Australia, and Asia [3]. Furthermore, *B. ceiba* is found throughout Thailand, especially in the north. Moreover, *B. ceiba* is an indigenous plant that is famous in the northern region of Thailand, and its stamen is considered an important ingredient in traditional Lanna food, such as “Nam Ngiao,” which is a curry soup served with rice noodles [4]. It is reported that this plant has been used in traditional medicine for diabetes, as an aphrodisiac, and to prevent cardiotoxicity [5,6,7]. Studies of ayurvedic traditional medicine have reported that most parts of *B. ceiba*, including the roots, leaves, gum, stem, and flowers, can prevent various diseases and improve health [8]. The flowers of *B. ceiba* have three cultivars, which are red, orange, and, rarely, white in color [9]. Thailand has the red and orange cultivars, and the orange stamen is the most popular and in high economic demand in the vegetable industry. In addition, these flowers have been used for anti-diabetic, antioxidant, antibacterial, and anti-Hepatitis B virus activities [2,10,11]. Previous studies have reported that *B. ceiba* flowers contain various bioactive components, such as quercetin, rutin, vitexin, isovitexin, kaempferol, isomangiferin, mangiferin, glucopyranoside, phenyl ethyl rutinoside, protocatechuic acid, chlorogenic acid, methylchlorogenate, and vanillic acid [12], which are phenolic and flavonoid groups. It was previously found that phenolics and flavonoids had antioxidant potential that enhanced the male reproductive system [13,14,15,16], but the *B. ceiba* stem bark, when extracted at a dose of 1000 mg/kg, had adverse effects on vital organs, including the heart, liver, and kidneys of mice [17]. However, there are limited scientific reports on the biological effects of *B. ceiba* stamens on the male reproductive system.

Reproductive disorders in males are causes of infertility and have a strong impact on population growth rates [18]. An imbalance of free radicals or reactive oxygen species (ROS), the elimination of free radicals, and the level of antioxidant capacity cause cell damage, resulting in male infertility [19]. Sperm abnormality is a cause of male reproductive dysfunction, which may result from a reduction in the ability of sperm macromolecules, including lipids and proteins [20]. Environmental toxins and chemical exposure contribute to male reproductive disorders and cause infertility [21]. Ferrous sulfate (Fe) is a chemical compound that can cause oxidative stress via superoxide radical formation [22]. Excessive exposure to Fe can induce oxidative stress and cause structural and functional abnormalities in spermatozoa [23].

The prevention and management of oxidative stress can improve male fertility capacity. Oxidative stress formation can be prevented by consuming sufficient antioxidants [21]. In recent years, phytochemical compounds from natural products have become popular in oxidative stress prevention and treatment [24]. Earlier studies reported that many medicinal plants, such as the rhizomes of *B. rotunda* [25,26], the rhizomes of *K. parviflora* [27], the leaves of *M. oleifera* [28], and the petals of *N. nucifera* [13,14], increased male sexual activity and sperm quality. These plants contain phenols and flavonoids. Therefore, natural antioxidants from *B. ceiba* stamens with phenols and flavonoids should be developed and used to prevent and manage oxidative stress and to enhance male reproductive function.

In Thailand, orange *B. ceiba* stamens are highly popular and are grown for traditional Lanna recipes. Consequently, the aims of this study are to investigate the phytochemical compounds of the orange *B. ceiba* stamen and its effect on the antioxidant properties and quality of cattle sperm treated with Fe.

## 2. Results

### 2.1. Phytochemical Contents

The total phenolic, total tannin, total flavonoid, total monomeric anthocyanin, and lycopene contents and yield of orange *B. ceiba* stamen hot aqueous extract (BAE), ultrasonicated extract (BUE), and microwave-assisted extract (BMAE) are presented in Table 1.

The orange BUE had significantly higher total phenolic and total tannin contents than the other extracts, while the total flavonoid content was significantly higher in the orange BAE than in the BUE and BMAE. Significantly, the orange BUE showed the highest total monomeric anthocyanin levels, followed by the BAE and BMAE. However, the lycopene content and percentage yield were not significantly different between any of the extracts (Table 1).

### 2.2. Phytochemical Screening Using Liquid Chromatography Quadrupole Time-of-Flight Mass Spectrometry (LC-QTOF-MS)

Using LC-QTOF-MS in negative mode, a qualitative analysis of the phytochemical screening related to the enhancements in the male reproductive system induced by the orange *B. ceiba* stamen extracts obtained via the three extraction methods was carried out. This information was used to match the ions’ scores with those in the database of MassHunter Qualitative Analysis Software version 10.0.

**Figure 1:**
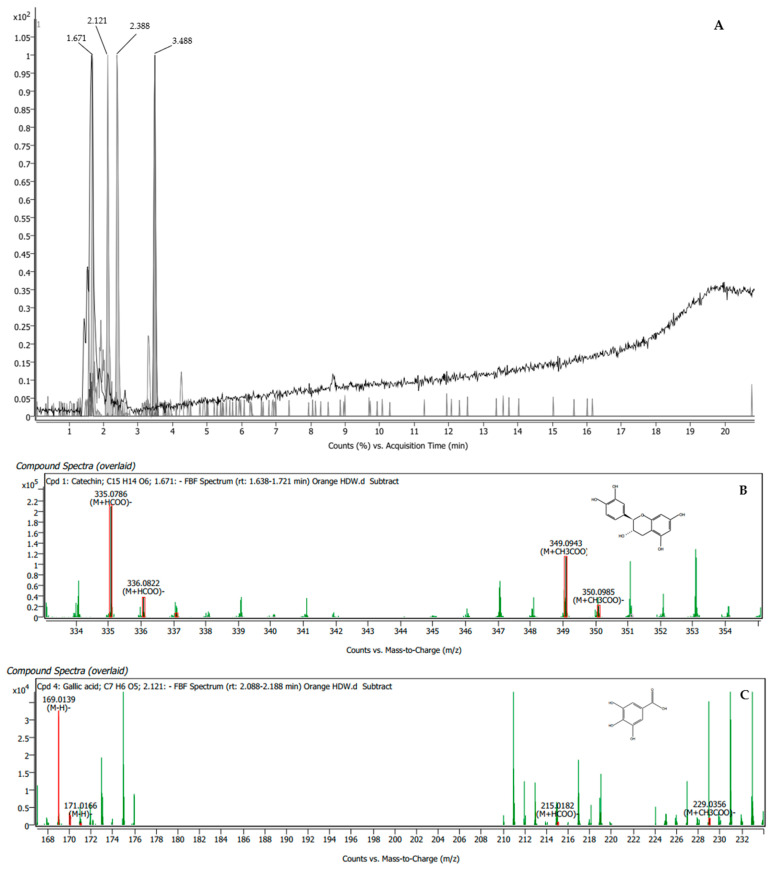

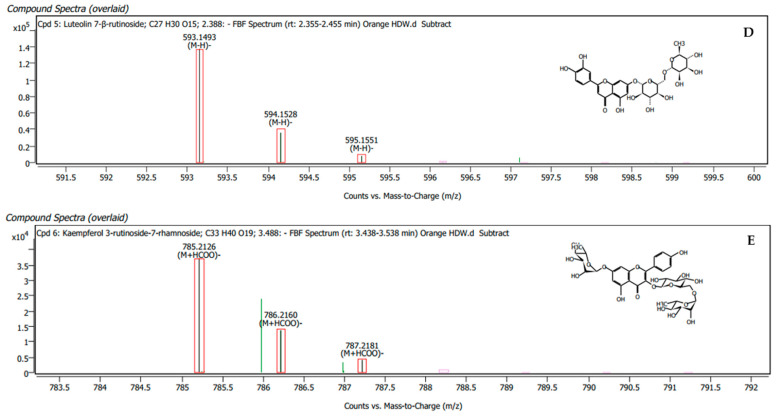
LC-QTOF/MS chromatogram of orange BAE showing peak identification of four phytochemical compounds related to male reproductive enhancement (**A**). Mass spectrum of catechin (**B**); gallic acid (**C**); luteolin 7-β-rutinoside (**D**); and kaempferol 3-rutinoside-7-rhamnoside (**E**).

The orange BAE was found to contain four compounds, namely catechin, gallic acid, luteolin 7-β-rutinoside, and kaempferol 3-rutinoside-7-rhamnoside, which presented scores of 93%, 92.11%, 91.18%, and 94.04%, respectively (Table 2). The specific peaks in the mass spectra and their corresponding compounds are displayed in Figure 1.

**Figure 2:**
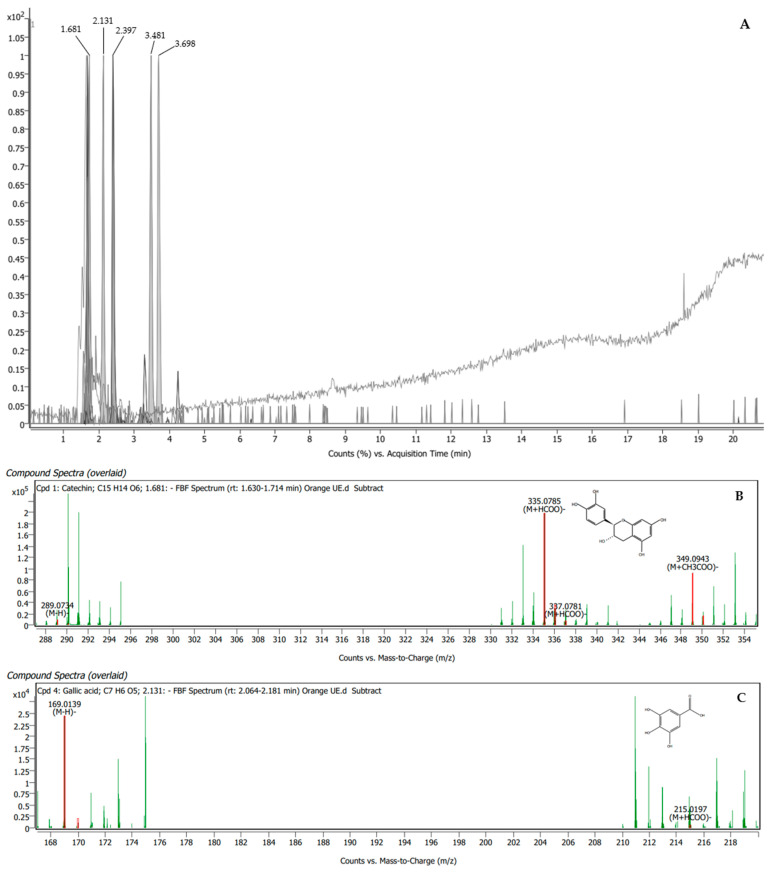

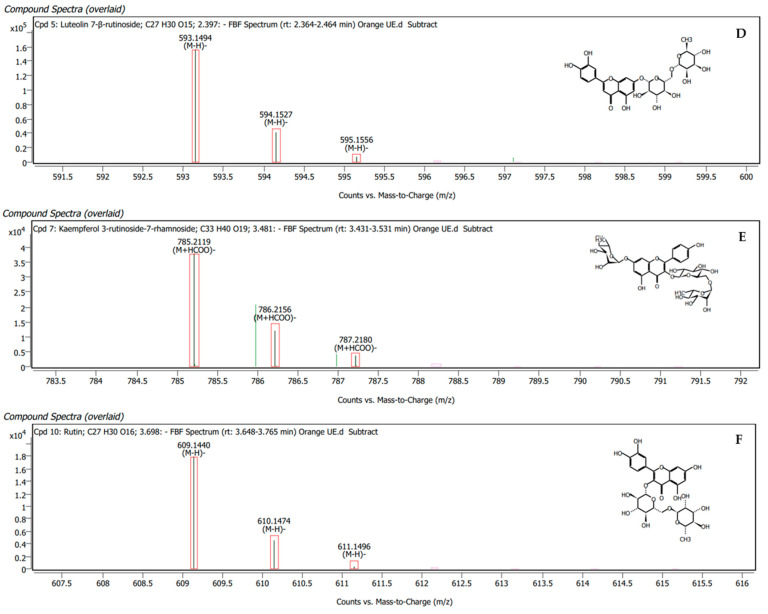
LC-QTOF/MS chromatogram of orange BUE showing peak identification of five phytochemical compounds related to male reproductive enhancement (**A**). Mass spectrum of catechin (**B**); gallic acid (**C**); luteolin 7-β-rutinoside (**D**); kaempferol 3-rutinoside-7-rhamnoside (**E**); and rutin (**F**).

The orange BUE was found to have five compounds, namely, catechin, gallic acid, luteolin 7-β-rutinoside, kaempferol 3-rutinoside-7-rhamnoside, and rutin, which presented matching scores of 90.51%, 83.14%, 92.07%, 87.96%, and 95.17%, respectively (Table 3). The specific peaks in the mass spectra and their corresponding compounds are displayed in Figure 2.

**Figure 3:**
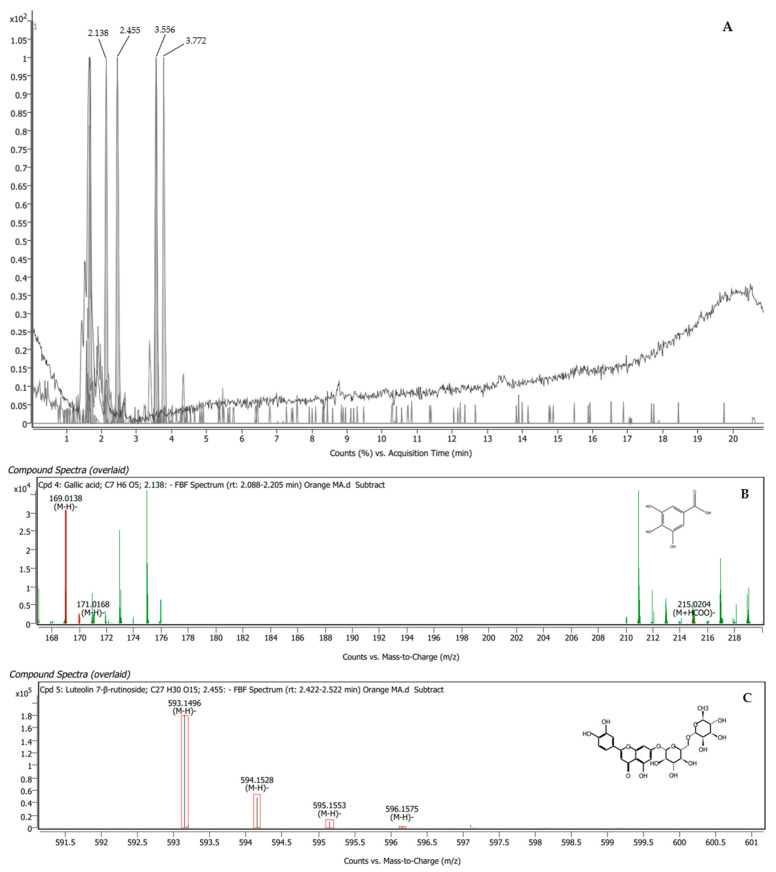

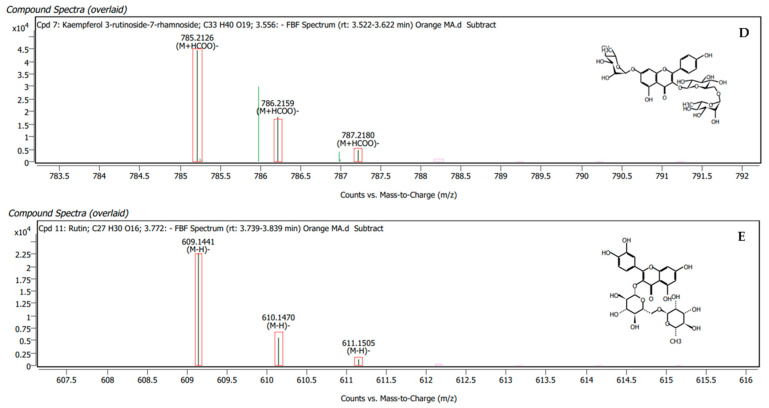
LC-QTOF/MS chromatogram of orange BMAE showing peak identification of four phytochemical compounds related to male reproductive enhancement (**A**). Mass spectrum of gallic acid (**B**); luteolin 7-β-rutinoside (**C**); kaempferol 3-rutinoside-7-rhamnoside (**D**); and rutin (**E**).

The orange BMAE was found to have four compounds, namely, gallic acid, luteolin 7-β-rutinoside, kaempferol 3-rutinoside-7-rhamnoside, and rutin, which presented matching scores of 98.47%, 93.31%, 92.77%, and 88.28%, respectively (Table 4). The specific peaks in the mass spectra and their corresponding compounds are displayed in Figure 3.

### 2.3. Antioxidant Properties in Cell-Free System

The half-maximal inhibitory concentration (IC50) values of 2,2-Diphenyl-1-picrylhydrazyl (DPPH), 2,2′-Azino-di-[3-Ethylbenzthiazoline sulfonate (ABTS), lipid peroxidation (LPO), and advance glycation end products (AGEs), and the ferric-reducing antioxidant power (FRAB) of orange *B. ceiba* stamen extracts obtained via different methods of extraction are presented in Table 5.

The orange BUE and BMAE had significantly lower IC50 concentrations of the 2,2′-Azino-di-[3-Ethylbenzthiazoline sulfonate (ABTS) radical scavenger than the BAE group. However, the orange BAE and BUE presented a significantly lower IC50 concentration of lipid peroxidation (LPO) than the BMAE group. Significantly, the orange BUE showed the highest concentration of total antioxidants, followed by the orange BMAE and BAE. The extraction methods were not significantly different in terms of DPPH free radical scavenging or AGE formation in the cell-free system model (Table 5).

### 2.4. Cytotoxicity Analysis

**Figure 4:**
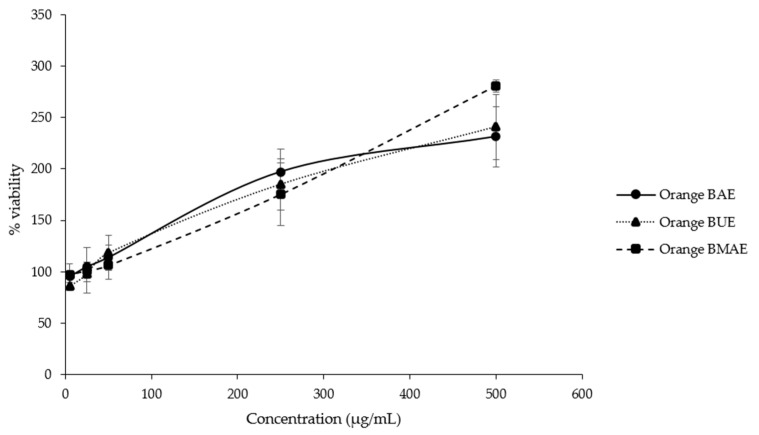
The percentage of sperm viability in controls treated with orange *B. ceiba* stamen extracts obtained via three different methods at doses of 5, 25, 50, 250, and 500 µg/mL. The data are displayed as the mean value ± standard deviation (error bar).

An MTT (3-[4,5-dimethylthiazol-2-yl]-2,5 diphenyl tetrazolium bromide) assay was carried out to evaluate cytotoxicity in the cattle sperm and to choose the appropriate concentration for a sperm quality analysis. In the controls treated with orange BAE, BUE, and BMAE, sperm viability was at least 95.20%, 86.06%, and 96.32%, respectively, indicating toxicity in the cattle sperm (Figure 4), and some concentrations enhanced sperm preservation more than the control.

### 2.5. Sperm Quality Analysis

After the cytotoxicity assay, the orange *B. ceiba* extracts at doses of 6.25, 12.5, 25, 50, and 100 µg/mL were used for cattle sperm quality analysis following the induction of oxidative stress with ferrous sulfate (Fe). In the sperm quality analysis, sperm motility, sperm morphology, sperm viability, and acrosome integrity were evaluated, and the results are presented below.

#### 2.5.1. Sperm Motility

Table 6 shows that the number of sperm exhibiting non-motility significantly increased in the Fe-treated group compared with in the control group. However, the groups treated with Fe and all concentrations of orange *B. ceiba* extracts, obtained via three different extraction methods, had a significantly decreased number of sperm exhibiting non-motility compared with the Fe group. The group treated with Fe and the orange BUE (25, 50, and 100 µg/mL) had a significantly decreased number of sperm exhibiting non-motility compared with the control and Fe groups. The number of progressive and non-progressive sperm exhibiting motility significantly decreased in the Fe-treated group compared with the control group. However, the groups treated with Fe and all orange *B. ceiba* extracts had a significantly increased number of progressive sperm exhibiting motility compared with the Fe-treated group. Moreover, in the group treated with the orange BUE (6.25 and 100 µg/mL), the number of progressive sperm exhibiting motility was significantly enhanced compared with the control and Fe groups. Moreover, the group treated with Fe and the orange BUE (25, 50, and 100 µg/mL) had a significantly increased number of non-progressive sperm exhibiting motility compared with the control and Fe groups. In contrast, the other groups had a significantly higher number of non-progressive sperm exhibiting motility than the Fe group only. The results show that a high concentration of the orange BUE had the greatest effect in terms of enhancing cattle sperm motility.

#### 2.5.2. Sperm Viability and Acrosome Integrity

**Figure 5:**
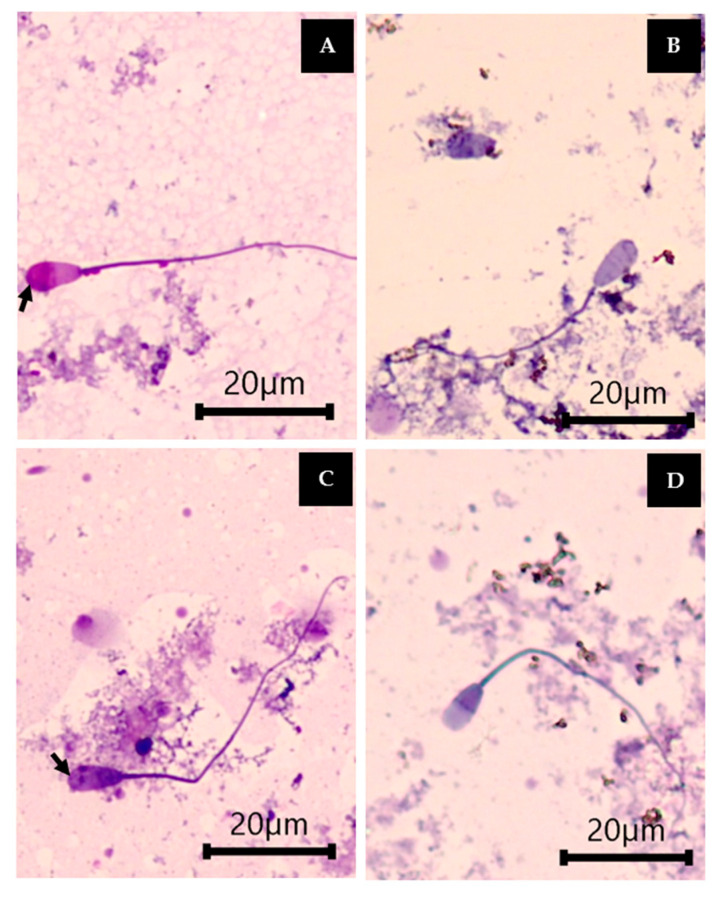
Charolais cattle sperm stained with TB/Giemsa for viability and acrosome integrity classification demonstrated as viable sperm with intact acrosomes (**A**), viable sperm with detached acrosomes (**B**), dead sperm with intact acrosomes (**C**), and dead sperm with detached acrosomes (**D**), shown at a magnification of 400×. The black arrow (*→*) indicates the acrosome of the cattle sperm.

The percentage of viable sperm with intact acrosomes significantly decreased in the Fe group, whereas the percentage of dead sperm with detached acrosomes significantly increased in the Fe group compared with the control. The groups treated with Fe and all orange *B. ceiba* extracts showed a significant decrease in the percentage of dead sperm with detached acrosomes compared with the Fe group, while in the group treated with the orange BUE at doses of 6.25, 12.5, and 50 µg/mL, this percentage was significantly decreased compared with the control and Fe groups. Nevertheless, the groups treated with Fe and the orange BAE (12.5, 25, and 50 µg/mL) and orange BUE (6.25 µg/mL) had a significantly decreased percentage of dead sperm with detached acrosomes compared with the control and Fe groups. Moreover, all orange *B. ceiba* extracts significantly increased the percentage of viable sperm with intact acrosomes compared with that in the Fe group, except for 50 µg/mL of the BUE and 100 µg/mL of the BMAE (Table 7). Therefore, the orange *B. ceiba* stamen extract obtained via decoction extraction has a high potential to improve cattle sperm viability. The Charolais cattle sperm viability and acrosome integrity are demonstrated in Figure 5.

#### 2.5.3. Sperm Morphology

The percentages of abnormal heads and tails significantly increased in the Fe-treated group compared with the control, while they were significantly decreased in the groups treated with Fe and all orange *B. ceiba* extracts, except for the orange BMAE (6.25, 12.5, 25, and 50 µg/mL), compared with the control and Fe groups. The groups treated with Fe and the orange BAE (all doses), orange BUE (12.5, 25, and 50 µg/mL), and orange BMAE (50 µg/mL) had a significantly decreased percentage of abnormal tails compared with the control and Fe groups. However, the percentage of normal sperm was significantly increased in the groups treated with Fe and the orange BAE (all doses), orange BUE (all doses), and orange BMAE (6.25, 50, and 100 µg/mL) compared with the control and Fe groups (Table 8).

### 2.6. Antioxidant Properties of Sperm

**Figure 6:**
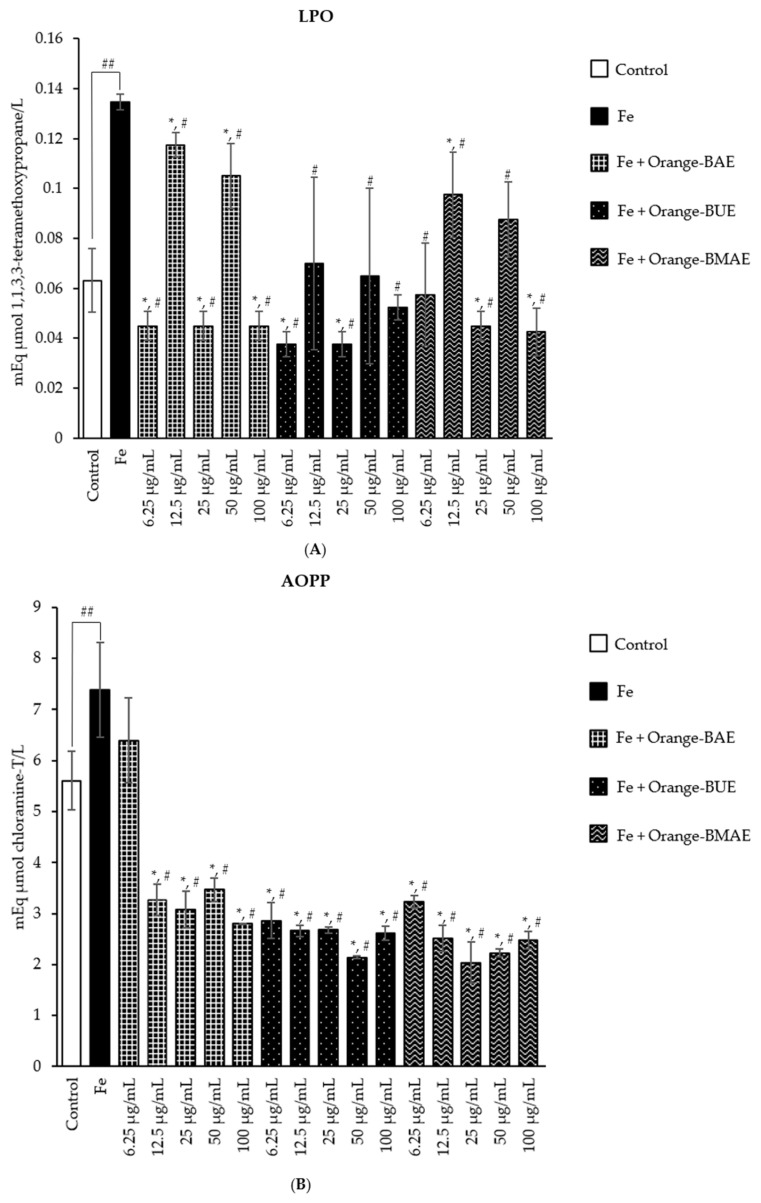

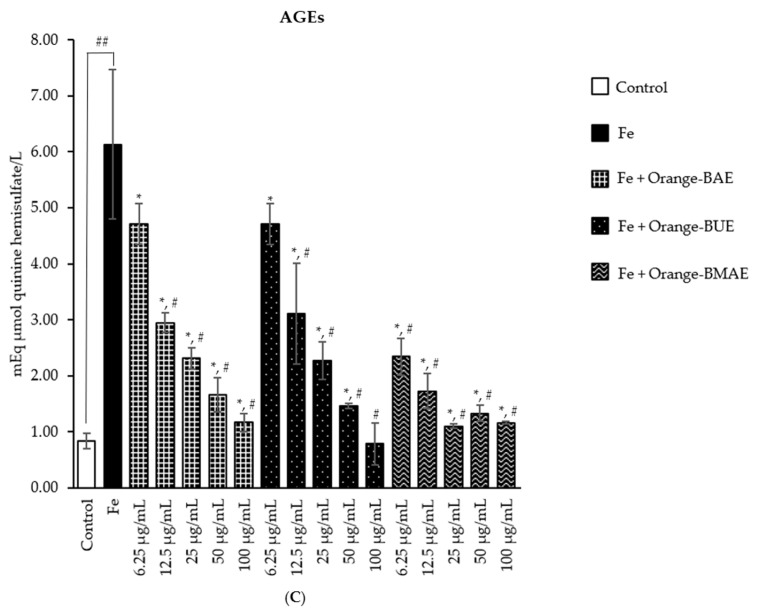
The mean value ± standard deviation (error bars) of LPO (**A**), AOPP (**B**), and AGEs (**C**) of cattle sperm samples treated as follows: control; Fe; Fe and orange BAE, orange BUE, and orange BMAE at doses of 6.25, 12.5, 25, 50, and 100 µg/mL (Fe: ferrous sulfate; BAE: *B. ceiba* stamen hot aqueous extract; BUE: *B. ceiba* stamen ultrasonicated extract; and BMAE: *B. ceiba* stamen microwave-assisted extract). Data were obtained from three replicates (*n* = 3). * indicates significant differences from the control group at *p* < 0.05; ^#^ indicates significant differences from the Fe group at *p* < 0.05; ^##^ indicates significant differences between the Fe and the control groups at *p* < 0.05.

The results of LPO, AOPP, and AGEs in cattle sperm treated with Fe only and with Fe and the orange *B. ceiba* extracts, obtained via three different extraction methods, are presented in Figure 6.

The concentration of LPO significantly increased in the sperm treated with Fe compared with the control group, while the concentration of LPO in the sperm treated with Fe and the orange BUE (12.5, 50, and 100 µg/mL) and orange BMAE (6.25 and 50 µg/mL) was significantly lower than in the Fe group and similar to that measured in the control. In addition, the concentration of LPO in the sperm treated with Fe and the orange BAE (6.25, 25, and 100 µg/mL), orange BUE (6.25 and 25 µg/mL), and orange BMAE (12.5 and 100 µg/mL) was significantly lower than in the Fe and control groups (Figure 6A).

The Fe-treated group showed a significant increase in the AOPP concentration compared with the control. The groups treated with Fe and all extracts presented a significant decrease in AOPP compared with the Fe and control groups, except for the orange BAE at a dose of 6.25 µg/mL, which presented results not significantly different from those of the Fe group (Figure 6B).

The formation of AGEs significantly increased in the sperm treated with Fe compared with the control. All *B. ceiba* stamen extract treatments significantly decreased the formation of AGEs in the Fe group, with 100 µg/mL of the orange BUE producing similar results to the control group (Figure 6C).

## 3. Discussion

This study used water as a solvent for all extraction methods because water is an efficient organic solvent for obtaining polyphenols from plant samples and is commonly used to extract aqua-soluble phytochemicals from herbal medicines [29]. Moreover, a previous report showed that aqueous extraction obtained higher amounts of phenolics and tannin compounds from plants than 95% ethanolic extraction [13]. The total phenolic content was determined via the reducing sugar reaction with the Folin–Ciocalteu reagent [30]. Phenolic compounds are polar and easily soluble by polar solvents [31]. This experiment found phenolic compounds following the use of all extraction methods because all methods used water as a solvent for orange *B. ceiba* stamen extraction, which is a popular green extraction method and plays an essential role in increasing phenolic solubility [32]. The total tannin content of the orange *B. ceiba* stamen extracts correlated with the total phenolic content, as tannins, phenolic compounds in plants, are soluble in water and organic solvents [33]. Moreover, the concentration of total monomeric anthocyanins presented similar variations in the total phenolic content because a sugar unit of anthocyanins is easily soluble in water and is found in plant pigments [34].

The present study used three extraction methods: decoction, ultrasonic extraction, and microwave-assisted extraction. Decoction is a traditional extraction method, and the procedure uses heat and boiling water, maintaining them for a particular duration to extract bioactive compounds from plant materials [35,36]. This is the most popular and well-known method in recipes and traditional medicine [29]. This study dissolved *B. ceiba* stamens in hot distilled water (75–80 °C) for 3–5 min. Ultrasonic extraction is a modern method that uses ultrasound energy and solvents to extract target compounds from natural products by increasing the movement speed and penetration force of the solvent’s molecules [37]. This method uses less time, less energy, and a low temperature for extraction [35]. The present study used an ultrasonic water bath at 50/60 Hz and 40 °C for 30 min. Additionally, microwave-assisted extraction is a modern method that uses electromagnetic energy to break the cell membrane of plant cells in order to extract active ingredients from within the plant cell [38]. This method can rapidly heat the extraction system, but it is limited to the factors required for proper operation in each type of plant extraction [39]; this study used 1 min of power on and 6 min of power off (heating to the desired temperature of about 70–80 °C).

The results show that the orange BUE contained the highest concentrations of total phenolic, total tannin, and total monomeric anthocyanin compounds, followed by the orange BAE and BMAE. In a previous study, the same ultrasonic extraction method preserved and increased the bioactive compound content in *Scutellaria barbata* more so than conventional extraction [40]. Polyphenols are a large group of phytochemicals that dissolve in organic solvents, with a wide melting temperature range across different extraction methods and durations [41]. A previous report stated that the maximum phenolic concentration is obtained at temperatures between 40 and 60 °C, while it declines at temperatures above 60 °C [42,43]. Moreover, the total monomeric anthocyanin compounds show maximum concentrations between 20 and 60 °C, whereas thermal degeneration occurs above 60 °C [41,44]. Therefore, the total phenolic, total tannin, and total monomeric anthocyanin compounds were found at the highest concentrations in the orange BUE. The reason for this might be that the temperature and duration used for ultrasonic extraction in this study produced ultrasound energy that increased the movement speed and penetration force of the solvent’s molecules, increasing the solubility of these compounds. However, the total phenolics, total tannins, and total monomeric anthocyanins of the orange BAE and BMAE might have been oxidized by the temperature and extraction methods used, resulting in degradation [34].

The results of the total flavonoid content showed the highest concentration in the orange BAE, followed by the BUE and BMAE. Similarly, in a previous study, a temperature of 80 °C resulted in the maximum total flavonoids in *Garcinia mangostana* [43]. Flavonoids are secondary metabolites from plants soluble in organic solvents, and they are more heat-sensitive [45]. This means that a temperature of 80 °C may be the most effective for flavonoid extraction.

LC-QTOF/MS of the orange BUE showed five compounds that affected male reproductive function, namely catechin, gallic acid, luteolin 7-β-rutinoside, kaempferol 3-rutinoside-7-rhamnoside, and rutin, while the BAE and BMAE showed four of these compounds, except for rutin and catechin, respectively. Consistently, the highest total phenolic content was found in the orange BUE. It is possible that ultrasonic extraction may be more effective for the extraction of high-quality and high quantities of polyphenols from orange *B. ceiba* stamens. Catechin, gallic acid, luteolin 7-β-rutinoside, kaempferol 3-rutinoside-7-rhamnoside, and rutin are flavonoids and phenolics, and they have been reported to enhance sperm characteristics, such as sperm motility, sperm viability, and plasma membrane and acrosome integrity, in in vitro and in vivo models [46,47,48,49,50,51]. This study demonstrates ultrasonic extraction as a good alternative for extracting polyphenols from orange *B. ceiba* stamens.

The antioxidant activity of the orange BAE, BUE, and BMAE in a cell-free system was determined using DPPH, ABTS, and FRAB assays. The orange BUE presented the most effectiveness in the ABTS and FRAB assays. Depending on how many phenolics, tannins, and anthocyanins are present, they act as natural antioxidant substances. The principle of an ABTS radical scavenging assay is based on a hydrogen donor for the antioxidant reaction, and the color of the reaction changes depending on the quantity of phytochemical compounds [52]. Moreover, the FRAP mechanism is based on electron transfer, which reduces Fe^3+^ to Fe^2+^ [31]. The orange BUE, which was extracted at low temperatures and used ultrasound energy to increase the solvent molecules’ movement for phytochemical compound extraction, is appropriate for eluting and extracting polyphenols, and it results in the highest number of polyphenols and the highest antioxidant capacity via hydrogen donor and electron transfer mechanisms.

Living cells have macromolecules, including lipids, proteins, and carbohydrates, which are free radical targets [21]. Living cell membranes contain unsaturated fatty acids, especially linoleic acid [20]. Therefore, this study used linoleic acid as the source of lipids in an LPO inhibition assay in a cell-free system. The orange BUE showed a significantly lower IC50 concentration of LPO than the BAE and BMAE. Phenolics and flavonoids have the potential to prevent LPO via free radical scavenging, resulting in cell damage prevention [24,53]. Significantly, the orange BUE had the highest phytochemical contents, resulting in decreased LPO formation, which manifests as a potent antioxidant ability.

The cytotoxic effects of the orange *B. ceiba* stamen extracts on cattle sperm were evaluated to determine the appropriate dose for an Fe toxicity test. All doses of the orange *B. ceiba* stamen extracts of all extraction methods showed no toxic effects on the Charolais cattle sperm. Moreover, a dose of 50 µg/mL or above could enhance sperm viability after thawing cattle spermatozoa. This shows that the orange *B. ceiba* stamen extract has no toxic effects and enhances sperm survival, especially at a dose of 50 µg/mL or above. In a previous study, after freezing and thawing semen, sperm viability decreased from environmental oxidative damage [54]. Moreover, this study used ferrous sulfate (Fe) to induce oxidative damage in cattle sperm, according to a previously used method for Fe-induced sperm damage [13,23]. Antioxidant agents were applied to prevent oxidative damage, thereby preserving sperm quality [47,51]. Therefore, this experiment was designed to examine the Fe-induced oxidative status and orange *B. ceiba* extract treatment for radical scavengers. The group treated with Fe exhibited decreased sperm motility and normal sperm morphology, sperm viability, and acrosome integrity. These results are similar to those of an in vitro study showing that Fe-induced rat sperm toxicity decreased sperm viability [13]. However, the orange *B. ceiba* extracts increased sperm motility, sperm viability, and sperm morphology compared to the Fe and normal control, especially the BUE and BAE. Moreover, this study found that all orange *B. ceiba* stamen extracts presented lower LPO and AGEs than the Fe-treated group. Living cell membranes containing unsaturated fatty acids, cholesterol, and glycosphingolipids are in direct contact with free radical particles, which are easily oxidized and fluctuate [21,55]. The LPO concentration of the sperm treated with Fe and the orange *B. ceiba* extract at 12.5 and 50 µg/mL was different from that of the sperm treated with 6.25, 25, and 100 µg/mL of all extracts obtained from different extraction methods; it is likely that the orange *B. ceiba* extracts are crude extracts. They consist of various phytochemical compounds, which have unique properties and specific mechanisms that have antioxidant effects at different concentrations [56]. Therefore, the orange *B. ceiba* extracts may cause fluctuating changes in the cell membrane and LPO levels. Moreover, the percentage of viable sperm with intact acrosomes slightly decreased after treatment with the orange *B. ceiba* extract at 12.5 and 50 µg/mL compared with treatment at the other doses. It is possible that these doses may affect the lipids, cholesterol, and glycosphingolipids of the cell membrane, causing cell membrane permeability [55] and decreased sperm viability and acrosome integrity. Future studies should fully elucidate these mechanisms. However, the orange *B. ceiba* extracts might have had a beneficial effect on free radical scavenging in the cattle sperm treated with Fe, resulting in cellular damage prevention [24]. Furthermore, the orange BUE had the greatest potential to enhance sperm quality, suggesting that the crude extract in the orange BUE contains a high amount of essential bioactive compounds that might have a direct effect on free radical scavenging in cattle sperm, resulting in the prevention of LPO and AGE formation. It is possible that the phytochemical compounds of the orange *B. ceiba* extracts had the potential to stimulate adenylate cyclase/cAMP/PKA signaling, stabilize the mitochondrial membrane, and protect the sperm membrane, leading to increased sperm motility and normal sperm morphology, sperm viability, and acrosome integrity [57]. Therefore, ultrasonic extraction is a good alternative method for preserving bioactive compounds from orange *B. ceiba* stamens and has the potential to effectively scavenge free radicals and increase sperm quality in in vitro models. Usually, thawed sperm is released to the outside before artificial insemination, resulting in reduced quality. Quality deterioration results from the freezing process and rapid deterioration after semen thawing [54]. Therefore, reducing the loss of sperm quality is necessary. From the results, it may be suggested that the orange BUE has benefits for sperm preservation and is attractive as an additive for the development of semen extenders. Although this study shows positive results from using the extracts for preserving antioxidant properties and sperm quality in vitro, it is possible that this extract may exert its mechanism of action on sperm, which may affect sperm fertilization. Moreover, the orange BUE is a good alternative substance for use as an additive in the development of semen extenders. This will need to be investigated in future studies.

## 4. Materials and Methods

### 4.1. Chemicals and Reagents

Folin–Ciocalteu reagent, sodium acetate, potassium acetate, 2,2-diphenyl-1-Picrylhydrazyl (DPPH), 2,20-Azino-di-(3-Ethylbenzthiazoline sulfonic acid) (ABTS), potassium hexacyanoferrate, thiobarbituric acid, glacial acetic acid, formic acid, and standard chemicals for calibration (including gallic acid, quercetin, tannic acid, tetramethoxypropane, chloramine-T, and quinine hemisulfate) were purchased from Sigma-Aldrich (St. Louis, MO, USA).

### 4.2. Plant Collection

**Figure 7:**
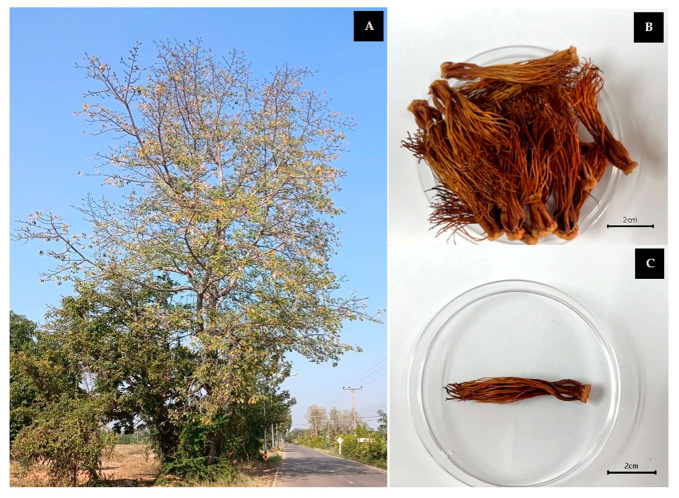
*Bombax ceiba* tree (**A**) and dried orange *Bombax ceiba* stamens (**B**,**C**).

Orange *B. ceiba* stamens were collected, and they originated from Thung Yang Subdistrict, Laplae District, Uttaradit Province, Thailand, located at 17°34′45.3″ N, 100°00′19.0″ E. The samples were deposited and authenticated at the Herbarium, Faculty of Pharmacy, Chiang Mai University, voucher number fl. 001. The *Bombax ceiba* tree and dried orange *Bombax ceiba* stamens are demonstrated in Figure 7.

### 4.3. Plant Extraction

The stamens were dried in light shade, pulverized, and stored at 4 °C before use. The dried stamens were extracted using three different methods:

1. The decoction method is a traditional extraction method used in recipes and traditional medicine. The *B. ceiba* stamen hot aqueous extract (BAE) was prepared by soaking dried samples in hot distilled water at 75–80 °C for 3–5 min [13];
2. Ultrasonic extraction is a modern method that uses ultrasound energy and solvents to extract target compounds from natural products. The *B. ceiba* stamen ultrasonic extract (BUE) was prepared by soaking dried samples in distilled water and subjecting them to ultrasonication in a water bath (Elma, Singen, Germany) at 50/60 Hz and 40 °C for 30 min [58];
3. Microwave-assisted extraction is a modern method that uses electromagnetic energy to break the cell membrane of plant cells. The *B. ceiba* stamen microwave-assisted extract (BMAE) was prepared by soaking dried samples in distilled water and pre-heating them using a magnetic stirrer at room temperature for 90 min. Then, suspensions were irradiated with microwaves (Milestone ETHOS UP, Sorisole, Italy) two times as follows: 1 min power on (heating to the desired temperature of about 70–80 °C) and 6 min power off [59].

**Figure 8:**
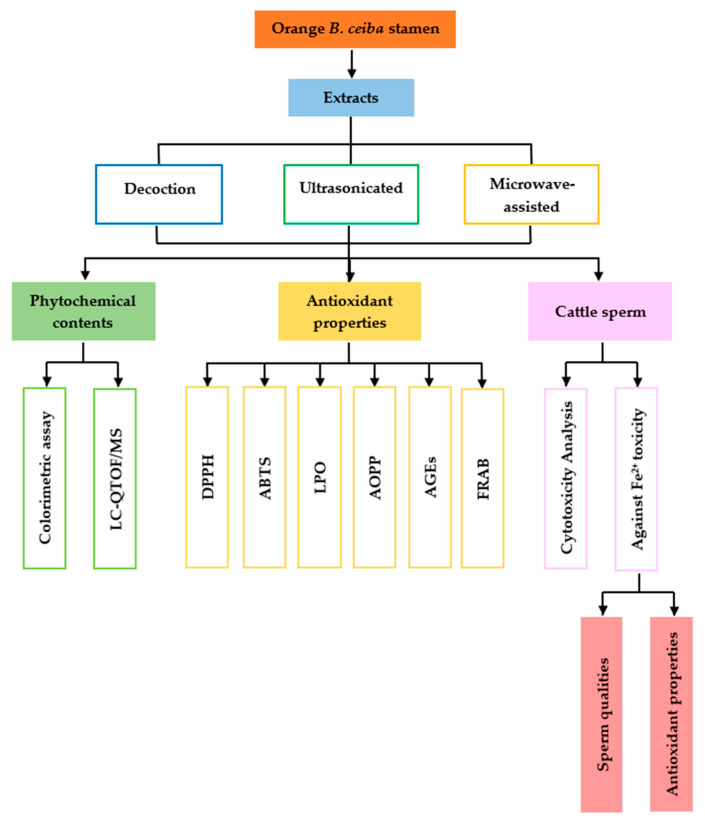
A schematic diagram showing the course of research activities.

After extraction, the solutions were filtrated and prepared for phytochemical and antioxidant activity analyses in a cell-free system with a 100 mg/mL stock concentration. Moreover, some parts of the extraction solutions were lyophilized and stored at −20 °C before sperm quality experimentation. The research activities are shown in a schematic diagram in Figure 8.

### 4.4. Phytochemical Contents

#### 4.4.1. Total Phenolic Content

The total phenolic content of the *B. ceiba* extracts was evaluated using the Folin–Ciocalteu reagent, and gallic acid was used as a standard. First, 200 µL of 1 M sodium carbonate (Na_2_CO_3_) was added to a mixture solution of 50 µL plant samples and 250 µL of 10% Folin–Ciocalteu reagent. The solutions were incubated at room temperature for 15 min, and the absorbance was measured at 765 nm using a microplate reader (BMG LABTECH, Offenburg, Germany). The total phenolic content was calculated as µg of gallic acid equivalents per g of plant dried weight [13].

#### 4.4.2. Total Flavonoid Content

A colorimetric assay was used for the determination of the total flavonoid content. A 50 µL solution of 10% aluminum chloride was added to a test tube containing 100 µL of the plant extracts. Then, 50 µL of potassium acetate and 700 µL of distilled water were added to the test tube, and the solutions were incubated at room temperature for 30 min. After incubation, 200 µL of each sample mixture was collected and placed into 96-well plates, and the absorbance was measured at 415 nm using a microplate reader (BMG LABTECH, Offenburg, Germany). The total flavonoid content was calculated as µg of quercetin equivalents per g of plant dried weight [13].

#### 4.4.3. Total Tannin Content

The total tannin content was determined using a Folin–Ciocalteu assay. First, 50 µL of the plant samples was added to a test tube containing 250 µL of 10% Folin–Ciocalteu reagent. Then, 200 µL of 1 M sodium carbonate (Na_2_CO_3_) was added and incubated at room temperature for 30 min. Next, 200 µL of each sample mixture was collected and placed into 96-well plates. Absorbance was measured at 700 nm using a microplate reader (BMG LABTECH, Offenburg, Germany). Tannic acid was used as the standard calibration, and the total tannin content was calculated as µg of tannic acid equivalents per g of plant dried weight [13].

#### 4.4.4. Total Monomeric Anthocyanin Content

The pH differential method was carried out to determine the total anthocyanin content [13]. First, 100 µL of plant extract was added to a test tube with 900 µL of 0.025 M potassium chloride buffer (pH 1.0) and 0.4 M sodium acetate buffer (pH 4.5). Then, these solutions were incubated at room temperature for 15 min, and 200 µL of each solution was collected and added to 96-well plates. The absorbance of each tube was measured at 510 nm and 700 nm using a microplate reader (BMG LABTECH, Offenburg, Germany). The absorbance of the diluted sample (A) was calculated as follows:

A = (A_510_ − A_700_)_pH 1.0_ − (A_510_ − A_700_)_pH 4.5_

The monomeric anthocyanin concentration in the solution was calculated using the following formula:

Monomeric anthocyanin pigment (µg/mL) = (A × MW × DF × 1000)/(ε × 1)

The anthocyanin content was calculated as cyanidin-3-glucoside when MW = 449.2 and ε = 26,900.

#### 4.4.5. Lycopene Content

Lycopene was extracted using a hexane–ethanol–acetone (2:1:1) (*v*/*v*) mixture [13]. First, 1 mL of DW was added to 0.5 g of each dried sample, vortexed, and incubated at 30 °C in a water bath for 60 min. Next, 4 mL of the mixture solution of hexane–ethanol–acetone (2:1:1) was added, vortexed, and incubated for 10 min in the dark. After incubation, 500 µL of DW water was added and vortexed, and the supernatants were collected and placed into 96-well plates. Absorbance was measured at 503 nm using a microplate reader (BMG LABTECH, Offenburg, Germany). The lycopene content was calculated according to the following formula:

Lycopene (mg/g plant dry weight) = A_503_ × 537 × 8 × 0.55/0.10 × 172

### 4.5. Phytochemical Screening of Extract Using LC-QTOF-MS

The phytochemical screening of the orange *B. ceiba* stamen extracts was analyzed using LC-MS with a ZORBAX Eclipse Plus C18 Rapid Resolution HD column (2.1 × 150 mm, 1.8 μm), utilizing a Liquid Chromatography Quadrupole Time-of-Flight Mass Spectrometry (LC-QTOF-MS) instrument (6545 LC/Q-TOF, Agilent Technology, Santa Clara, CA, USA), which consisted of a binary pump. The samples were dissolved in 70% methanol at a concentration of 1 mg/mL and passed through a 0.22 µm filter. The temperature was maintained at 35 °C, and the injection volume was 1 μL. Elution was performed for 21 minutes with a mobile-phase gradient system starting with 20% ACN and 80% water (0.1% formic acid), with a flow rate of 0.2 mL/min. The MS conditions involved an electrospray ionization (ESI) probe in the negative mode. MassHunter Qualitative Analysis Software (Agilent MassHunter Workstation Qualitative Analysis version 10.0, CA, USA) was used to evaluate the ion’s match with the database, and this was carried out by comparing the measured mass to the exact mass of the molecular formula and the predicted isotope pattern, utilizing the find-by-formula search of the Agilent MassHunter METLIN Metabolomics Database. The chemical characteristics of the compounds were obtained using the Medlin library [60].

### 4.6. Antioxidant Properties in Cell-Free System

#### 4.6.1. 2,2-Diphenyl-1-Picrylhydrazyl (DPPH) Radical Scavenging Assay

The free radical scavenging activity of the *B. ceiba* extracts was determined using a DPPH radical scavenging assay. In brief, 50 µL of various concentrations of each *B. ceiba* extract was added to 200 µL of 0.004% DPPH solution in methanol. The solution was incubated at room temperature for 30 min under dark conditions, and gallic acid was used as a positive control. Absorbance was measured at 515 nm using a microplate reader (BMG LABTECH, Offenburg, Germany), and the results were calculated as the half-maximal inhibition concentration [13].

#### 4.6.2. 2,2′-Azino-di-[3-Ethylbenzthiazoline Sulfonate (ABTS) Radical Scavenging Assay

ABTS was used to determine the free radical scavenging activity. ABTS was prepared with distilled water and stored in the dark at 4 °C. The stock solution was diluted to achieve an absorbance of 0.7 at 734 nm. Then, 50 µL of the various concentrations of each *B. ceiba* extract was added to 200 µL of ABTS working solution and incubated at room temperature for 30 min under dark conditions. Gallic acid was used as a positive control, and the absorbance was measured at 515 nm using a microplate reader (BMG LABTECH, Offenburg, Germany). The results were calculated as the half-maximal inhibition concentration [13].

#### 4.6.3. Lipid Peroxidation (LPO) Assay

A thiobarbituric acid reactive substance (TBARS) assay was used to determine the inhibition of lipid peroxidation. First, 50 µL of different concentrations of each *B. ceiba* extract was added to 10 mM linoleic acid emulsion in phosphate buffer at pH 7.4, and LPO was induced by 0.07 M FeSO_4_. Then, 100 µL distilled water was added to the mixture solution and incubated at room temperature for 15 min under dark conditions. After incubation, 225 µL of 0.85% normal saline solution, 500 µL 10% TCA, and 100 µL of thiobarbituric acid (TBA) were added and boiled at 95 °C for 30 min. The solutions were cooled by placing them at room temperature, followed by centrifugation at 3500 rpm for 5 min. The supernatant was collected and measured at 532 nm with a microplate reader (BMG LABTECH, Offenburg, Germany). The results were calculated as the half-maximal inhibition concentration [13].

#### 4.6.4. Inhibition of Formation of Advance Oxidation Protein Products (AOPPs)

First, 50 µL of different concentrations of each *B. ceiba* extract was added to 1 mg/mL of bovine serum albumin (BSA) (100 µL, in 0.2 M PBS, pH: 7.4), and protein oxidation was induced by 15 µL of 0.07 M of FeSO_4_. The mixture solutions were incubated at room temperature for 30 min under dark conditions. Then, 50 µL of 1.16 M potassium iodide (KI) was added, followed by 2 min of incubation. The solution was measured at 340 nm with a microplate reader (BMG LABTECH, Offenburg, Germany), and the results were calculated as the half-maximal inhibition concentration [13].

#### 4.6.5. Inhibition of Formation of Advance Glycation End Products (AGEs)

The inhibition of the AGEs of the *B. ceiba* extract was measured according to a previous study. First, 50 µL of different concentrations of each *B. ceiba* extract was added to 50 µL of BSA, and AGEs were induced by 50 µL of 1 M of D-glucose. The mixture solutions were incubated at 50 °C for 24 h under dark conditions. The fluorescence intensity (with an excitation wavelength of 360 nm and an emission wavelength of 460 nm) was measured using a microplate reader (Perkin Elmer, Singapore), and the results were calculated as the half-maximal inhibition concentration [13].

#### 4.6.6. Ferric-Reducing Antioxidant Power (FRAP) Assay

An FRAB assay was used for the determination of total antioxidants, which is measured as an absorbance change in the ferrous TPTZ complex. The FRAP reagent was prepared by mixing 100 mL of 300 mM acetate buffer (pH 3.6), 10 mL of 10 mM TPTZ solution, and 10 mL of 20 mM FeCl_3_ solution. Then, 200 µL of the FRAP reagent was added to a 96-well plate with 50 µL of the plant extracts and incubated at 37 °C for 4 min. Gallic acid was used as the standard, and the absorbance was measured at 593 nm using a microplate reader (BMG LABTECH, Offenburg, Germany). The result was calculated as mEq µmol gallic acid/L [13].

### 4.7. Cytotoxicity Analysis

Frozen Charolais cattle semen straws were purchased from Namchuea Wongwi Company Ltd. (Chiang Mai, Thailand). The frozen semen was thawed and centrifuged at 2500 rpm for 5 min. The sperm pellet was washed in triplicate with Krebs media (pH 7.4), and the concentration was adjusted to 10 × 10^6^ sperm/mL. Five concentrations (500, 250, 50, 25, 5 µg/mL) of the orange BAE, BUE, and BMAE were added to 96-well plates containing 100 µL of the sperm suspension. The mixture solution was incubated at 37 °C for 3 h, and then sperm viability was determined using an MTT viability assay. The results were calculated for each concentration as a percentage of the control, and appropriate concentrations were used for Fe toxicity studies [14].

### 4.8. Experimental Design

The frozen cattle sperm was prepared in Krebs media at 10 × 10^6^ sperm/mL. The treatments were separated as follows: Group I = Krebs solution (control); Group II = Fe 20 µg/mL; Groups III–VII = Fe 20 + orange BAE at 6.25, 12.5, 25, 50, and 100 µg/mL, respectively; Groups VIII–XII = Fe 20 + orange BUE at 6.25, 12.5, 25, 50, and 100 ug/mL, respectively; and Groups XIII–XVII = Fe 20 + orange BMAE at 6.25, 12.5, 25, 50, and 100 µg/mL, respectively. All treatments were added to separate test tubes containing 500 µL of the sperm suspension and incubated at 37 °C for 3 h. The experiment was conducted in three replicates. After incubation, the mixture solution was centrifuged at 2500 rpm for 5 min to separate the supernatant and sperm pellet.

### 4.9. Analysis of Sperm Characteristics

#### 4.9.1. Sperm Motility

Sperm motility was analyzed under a light microscope (Olympus CX31, Olympus Corporation, Tokyo, Japan) at a magnification of 400×. Briefly, 20 µL of the mixed sperm solution in each test tube was dropped into an improved Neubauer hemocytometer and video-recorded. A total of 200 sperm were counted and classified into four patterns per test tube: sperm non-motility, sperm progressive motility, circle motility, and non-progressive motility [14].

#### 4.9.2. Sperm Viability and Acrosome Integrity

The sperm solution in each test tube was mixed with trypan blue (TB) (1:1 *v*/*v*) and placed and smeared on a slide. Each slide was air-dried and fixed in 0.2% of a neutral red fixative solution, which was dissolved in a mixture solution of 86 mL of 1N HCL and 14 mL of 37% formaldehyde solution, for 4 min. The slide was rinsed with tap and distilled water after being stained with 7.5% Giemsa solution at 40 °C for 4 h. It was subsequently air-dried and covered by a cover slit for imaging under a light microscope at a magnification of 400×. A total of 100 sperm per test tube were classified in terms of sperm viability and acrosome integrity [14].

#### 4.9.3. Sperm Morphology

The photograph taken under a light microscope at 400× magnification of the sperm stained with TB/Giemsa was used for morphology determination. A total of 100 sperm per test tube were classified in terms of sperm morphology [14].

### 4.10. Antioxidant Properties in Sperm

#### 4.10.1. Lipid Peroxidation (LPO) Assay

The lipid peroxidation assay of the sperm was investigated using a thiobarbituric acid-reactive species (TBARS) assay. Briefly, 225 µL of 0.85% normal saline solution, 500 µL 10% TCA, and 100 µL of TBA were added to a test tube containing 100 µL sperm supernatant. Then, the mixture solution was boiled at 95 °C for 30 min and placed at room temperature for cooling. The solutions were centrifuged at 3500 rpm for 5 min, and the supernatant was collected and placed in 96-well plates. The supernatant was measured at 532 nm with a microplate reader (BMG LABTECH, Offenburg, Germany). The compound 1,1,3,3-tetramethoxypropane (TMP) was used for standard calibration, and the result was calculated and is expressed as mEq µmol 1,1,3,3-tetramethoxypropane/L [14].

#### 4.10.2. Inhibition of Formation of Advance Oxidation Protein Products (AOPPs)

The determination of AOPP formation was investigated according to the method in a cell-free system by using 100 µL of supernatant in each test tube. The standard calibration curve was obtained using chloramine-T, and the result is expressed as mEq µmol chloramine-T/L [14].

#### 4.10.3. Inhibition of Formation of Advance Glycation End Products (AGEs)

First, 100 µL of supernatant was added to a 96-well plate, and AGE formation was determined using a microplate reader at an excitation wavelength of 360 nm and an emission wavelength of 460 nm (Perkin Elmer, Singapore). The standard calibration curve was obtained using quinine hemisulfate, and the result is expressed as mEq µmol quinine hemisulfate/L [14].

### 4.11. Statistical Analysis

The data are displayed as the mean ± standard deviation (SD). Excel Microsoft 365 was used to determine the half-maximal inhibition concentration (IC50). All experiments were carried out in three replicates, and the significance level was set at *p* < 0.05. The normal distribution of phytochemical and antioxidant activities in cell-free systems was analyzed using the Shapiro–Wilk test, while other parameters were determined using the Kolmogorov–Smirnov test. The statistical analysis of the mean values of the phytochemical content and antioxidant activity in cell-free systems was carried out using a one-way ANOVA, followed by Duncan’s tests to analyze the differences between groups. The independent *t*-test or Mann–Whitney U test was used for a comparison between MZ and control groups. The Kruskal–Wallis and Mann–Whitney U tests were performed to analyze the differences between groups of mean values of other parameters.

## 5. Conclusions

In conclusion, the orange *B. ceiba* stamen is rich in polyphenols and has antioxidant potential in cell-free systems. The orange BUE had the highest phytochemical content and effective antioxidant activity. The orange BUE enhanced sperm motility, and both the orange BUE and BAE enhanced sperm viability and normal sperm morphology by scavenging free radicals and increasing antioxidants. From these results, it may be suggested that ultrasonic extraction has benefits for phytochemical preservation in *B. ceiba* stamens. The orange BUE has benefits for sperm preservation and increasing the economic value of the local plants. The orange BUE is a good alternative substance for use as an additive in semen extenders, and this should be further studied.

## Figures and Tables

**Table 1:** Total phenolic, total tannin, total flavonoid, total monomeric anthocyanin, and lycopene contents and yield of orange *B. ceiba* stamens using different extraction methods.

Sample	Extraction	Total Phenolics (µg GAE/g Dried Weight)	Total Tannins (µg TAE/g Dried Weight)	Total Flavonoids (µg QE/g Dried Weight)	Total Monomeric Anthocyanins (µg Cyanidin-3-glucoside E/g Dried Weight)	Lycopene Content (×10^2^ mg/g Dried Weight)	Yield (%)
*B. ceiba*	Decoction	4.20 ± 0.30 ^a^	3.55 ± 0.26 ^a^	0.26 ± 0.02 ^b^	6.29 ± 0.42 ^a^	23.84 ± 22.76	10.99 ± 4.01
	Ultrasonicated	5.03 ± 0.14 ^b^	4.29 ± 0.11 ^b^	0.13 ± 0.04 ^a^	7.51 ± 0.29 ^b^	21.44 ± 9.32	10.78 ± 4.18
	Microwave-assisted	4.11 ± 0.10 ^a^	3.46 ± 0.03 ^a^	0.10 ±0.05 ^a^	5.18 ± 0.44 ^c^	20.73 ± 6.95	10.82 ± 3.65

Results are presented as the mean ± SD (*n = 3*). ^a,b,c^ Different letters indicate significant differences between groups in column data at *p* < 0.05. All parameters were analyzed using one-way ANOVA, followed by Duncan’s test.

**Table 2:** Compounds identified in orange *B. ceiba* stamen extract obtained using the aqueous decoction method (orange BAE) according to LC-QTOF-MS.

No.	Compound	Formula	RT	Matching Score (%)	*m/z*	Mass	Mass Diff (db/ppm)
1	Catechin	C_15_ H_14_ O_6_	1.671	93.00	335.0786	290.0805	5.11
2	Gallic acid	C_7_ H_6_ O_5_	2.121	92.11	169.0139	170.0215	−2.90
3	Luteolin 7-β-rutinoside	C_27_ H_30_ O_15_	2.388	91.81	593.1493	594.1566	−3.20
4	Kaempferol 3-rutinoside-7-rhamnoside	C_33_ H_40_ O_19_	3.488	94.04	785.2126	740.2143	−2.75

**Table 3:** Compounds identified in orange *B. ceiba* stamen extract obtained using the aqueous ultrasonication method (orange BUE) according to LC-QTOF-MS.

No.	Compound	Formula	RT	Matching Score (%)	*m/z*	Mass	Mass Diff (db/ppm)
1	Catechin	C_15_ H_14_ O_6_	1.681	90.51	335.0785	290.0802	4.16
2	Gallic acid	C_7_ H_6_ O_5_	2.131	83.14	169.0139	170.0212	−1.62
3	Luteolin 7-β-rutinoside	C_27_ H_30_ O_15_	2.397	92.07	593.1494	594.1567	−2.97
4	Kaempferol 3-rutinoside-7-rhamnoside	C_33_ H_40_ O_19_	3.481	87.96	785.2119	740.2138	−3.50
5	Rutin	C_27_ H_30_ O_16_	3.698	85.17	609.1440	610.1513	−3.41

**Table 4:** Compounds identified in orange *B. ceiba* stamen extract obtained using the aqueous microwave-assisted method (orange BMAE) according to LC-QTOF-MS.

No.	Compound	Formula	RT	Matching Score (%)	*m/z*	Mass	Mass Diff (db/ppm)
1	Gallic acid	C_7_ H_6_ O_5_	2.138	98.47	169.0138	170.0211	−2.65
2	Luteolin 7-β-rutinoside	C_27_ H_30_ O_15_	2.455	93.31	593.1496	594.1568	−2.80
3	Kaempferol 3-rutinoside-7-rhamnoside	C_33_ H_40_ O_19_	3.556	92.77	785.2126	740.2144	−2.73
4	Rutin	C_27_ H_30_ O_16_	3.772	88.28	609.1441	610.1513	−3.43

**Table 5:** The half-maximal inhibitory concentration (IC50) values of DPPH, ABTS, LPO, and AGEs, and the total antioxidants (FRAB) of orange *B. ceiba* stamen extracts obtained using different extraction methods.

Sample	Extraction	IC50 (mg/mL)				FRAB (mEq µmol Gallic Acid/L)
		DPPH	ABTS	LPO	AGEs	
*B. ceiba*	Decoction	0.48 ± 0.02	0.94 ± 0.15 ^a^	2.34 ± 0.12 ^a^	33.59 ±0.97	9.97 ± 0.28 ^a^
	Ultrasonicated	0.45 ± 0.05	0.54 ± 0.08 ^b^	1.36 ± 0.70 ^a^	35.35 ± 1.97	12.95 ± 0.18 ^b^
	Microwave-assisted	0.45 ± 0.06	0.62 ± 0.06 ^b^	7.30 ± 1.66 ^b^	30.35 ± 5.13	11.54 ± 0.50 ^c^

Results are presented as the mean ± SD (*n = 3*). ^a,b,c^ Different letters indicate significant differences between groups in column data at *p* < 0.05. All parameters were analyzed using one-way ANOVA, followed by Duncan’s test.

**Table 6:** The number of sperm exhibiting motility and non-motility of Charolais cattle sperm samples were treated with ferrous sulfate (Fe) and orange *B. ceiba* extract obtained via different extraction methods, and the control group.

Group	Concentration (µg/mL)	Number of Sperm Exhibiting Motility			Number of Sperm Exhibiting Non-Motility
		Progressive	Circle	Non-Progressive	
**Control**		3.33 ± 0.58	3.33 ± 2.08	31.67 ± 0.58	161.67 ± 1.53
**Ferrous sulfate (Fe)**	20 µg/mL	0.00 ± 0.00 ^##^	0.00 ± 0.00 ^##^	0.33 ± 0.58 ^##^	199.67 ± 0.58 ^##^
**Fe + orange BAE**	6.25 µg/mL	4.33 ± 3.06 ^#^	0.00 ± 0.00 *	30.67 ± 9.61 ^#^	165.00 ± 12.53 ^#^
	12.5 µg/mL	1.33 ± 0.58 *^,#^	0.00 ± 0.00 *	33.33 ± 7.7 ^#^	165.33 ± 8.14 ^#^
	25 µg/mL	4.00 ± 2.00 ^#^	0.00 ± 0.00 *	40.67 ± 15.04 ^#^	155.33 ± 16.86 ^#^
	50 µg/mL	2.00 ± 0.01 *^,#^	0.00 ± 0.00 *	30.00 ± 3.00 ^#^	168.00 ± 3.00 *^,#^
	100 µg/mL	2.33 ± 1.53 ^#^	0.00 ± 0.00 *	30.67 ± 12.22 ^#^	167.00 ± 13.75 ^#^
**Fe + orange BUE**	6.25 µg/mL	5.67 ± 0.58 *^,#^	0.00 ± 0.00 *	29.67 ± 1.53 ^#^	164.67 ± 2.08 ^#^
	12.5 µg/mL	3.67 ± 0.58 ^#^	0.00 ± 0.00 *	32.00 ± 2.65 ^#^	164.33 ± 3.06 ^#^
	25 µg/mL	3.67 ± 0.58 ^#^	0.00 ± 0.00 *	41.33 ± 1.53 *^,#^	155.00 ± 1.00 *^,#^
	50 µg/mL	7.00 ± 3.00 ^#^	0.00 ± 0.00 *	68.33 ± 13.50 *^,#^	124.67 ± 16.50 *^,#^
	100 µg/mL	8.00 ± 1.00 *^,#^	0.00 ± 0.00 *	82.33 ± 22.50 *^,#^	109.67 ± 23.50 *^,#^
**Fe + orange BMAE**	6.25 µg/mL	1.33 ± 0.58 *^,#^	0.00 ± 0.00 *	35.00 ± 4.36 ^#^	163.67 ± 4.62 ^#^
	12.5 µg/mL	1.00 ± 0.01 *^,#^	0.00 ± 0.00 *	24.00 ± 1.00 *^,#^	175.00 ± 1.00 *^,#^
	25 µg/mL	0.00 ± 0.00 *	0.00 ± 0.00 *	15.33 ± 0.58 *^,#^	184.67 ± 0.58 *^,#^
	50 µg/mL	1.67 ± 0.58 *^,#^	0.00 ± 0.00 *	29.00 ± 1.00 *^,#^	169.33 ± 1.53 *^,#^
	100 µg/mL	1.00 ± 0.01 *^,#^	0.00 ± 0.00 *	31.67 ± 3.06 ^#^	167.33 ± 3.06 *^,#^

The number of sperm exhibiting motility and non-motility was analyzed using the Kruskal–Wallis test, followed by the Mann–Whitney U test. Data were obtained from nine replicates (*n* = 3). * indicates significant differences from the control group at *p* < 0.05; ^#^ indicates significant differences from the Fe group at *p* < 0.05; ^##^ indicates significant differences between the Fe and the control groups at *p* < 0.05.

**Table 7:** Percentages of sperm viability (viable and dead) and acrosome integrity (intact and detached acrosomes) of Charolais cattle sperm samples treated with ferrous sulfate (Fe) and orange *B. ceiba* extracts obtained via different extraction methods and the control group.

Group	Concentration (µg/mL)	Viable Sperm (%)		Dead Sperm (%)	
		Intact	Detached	Intact	Detached
**Control**		31.00 ± 6.00	28.00 ± 3.00	13.67 ± 5.51	27.33 ± 8.50
**Ferrous sulfate (Fe)**	20 µg/mL	4.00 ± 1.00 ^##^	23.00 ± 5.00	15.00 ± 10.54	58.00 ± 6.56 ^##^
**Fe + orange BAE**	6.25 µg/mL	36.00 ± 3.00 ^#^	40.67 ± 0.58 *^,#^	11.00 ± 1.00	12.33 ± 3.51 *^,#^
	12.5 µg/mL	29.33 ± 5.51 ^#^	39.33 ± 18.50	16.33 ± 8.50	15.00 ± 4.58 ^#^
	25 µg/mL	34.67 ± 2.52 ^#^	36.00 ± 1.00 *^,#^	16.33 ± 1.53	13.00 ± 2.00 *^,#^
	50 µg/mL	19.33 ± 2.52 *^,#^	50.00 ± 5.00 *^,#^	16.67 ± 1.53	14.00 ± 1.00 *^,#^
	100 µg/mL	30.67 ± 3.51 ^#^	29.00 ± 6.00	23.33 ± 1.53 *	17.00 ± 4.00 ^#^
**Fe + orange BUE**	6.25 µg/mL	38.00 ± 18.00 ^#^	25.00 ± 4.00	30.00 ± 18.00	7.00 ± 4.00 *^,#^
	12.5 µg/mL	24.67 ± 8.50 ^#^	30.00 ± 5.00	25.00 ± 2.00 *	20.33 ± 1.53 ^#^
	25 µg/mL	19.00 ± 2.65 *^,#^	39.33 ± 26.50	23.00 ± 15.00	18.67 ± 9.07 ^#^
	50 µg/mL	20.67 ± 17.50	40.00 ± 17.00	20.33 ± 11.50	19.00 ± 12.00 ^#^
	100 µg/mL	26.67 ± 10.02 ^#^	25.33 ± 13.50	30.00 ± 12.00	18.00 ± 8.54 ^#^
**Fe + orange BMAE**	6.25 µg/mL	8.67 ± 3.51 *	52.00 ± 22.00 ^#^	22.00 ± 16.52	17.33 ± 2.31 ^#^
	12.5 µg/mL	25.67 ± 6.51 *^,#^	37.33 ± 7.51	18.00 ± 10.00	19.00 ± 4.00 ^#^
	25 µg/mL	14.67 ± 1.53 *^,#^	26.00 ± 8.00	30.67 ± 10.50 *	28.67 ± 4.04 ^#^
	50 µg/mL	19.00 ± 4.00 *^,#^	38.33 ± 11.50	18.67 ± 2.52	24.00 ± 5.00 ^#^
	100 µg/mL	13.67 ± 11.50	52.00 ± 7.00 *^,#^	6.00 ± 1.00 *	28.33 ± 5.51 ^#^

Percentages of sperm viability and acrosome integrity were analyzed using the Kruskal–Wallis test, followed by the Mann–Whitney U test. Data were obtained from nine replicates (*n* = 3). * indicates significant differences from the control group at *p* < 0.05; ^#^ indicates significant differences from the Fe group at *p* < 0.05; ^##^ indicates significant differences between Fe and the control groups at *p* < 0.05.

**Table 8:** Percentages of normal and abnormal Charolais cattle sperm in the groups treated with ferrous sulfate (Fe) and orange *B. ceiba* extracts, obtained via different extraction methods, and in the control group.

Group	Concentration (µg/mL)	Normal Sperm (%)	Abnormal Sperm (%)		
			Head Only	Head and Tail	Tail Only
**Control**		45.33 ± 5.51	2.67 ± 0.58	6.33 ± 1.53	45.67 ± 3.51
**Ferrous sulfate (Fe)**	20 µg/mL	36.00 ± 6.00	6.00 ± 4.00	17.67 ± 6.51 ^##^	40.33 ± 4.51
**Fe + orange BAE**	6.25 µg/mL	64.67 ± 2.52 *^,#^	2.00 ± 2.00	0.67 ± 0.58 *^,#^	32.67 ± 5.03 *^,#^
	12.5 µg/mL	72.67 ± 9.50 *^,#^	0.33 ± 0.58 *^,#^	0.67 ± 0.58 *^,#^	26.33 ± 8.50 *^,#^
	25 µg/mL	77.00 ± 2.00 *^,#^	0.00 ± 0.00 *^,#^	0.00 ± 0.00 *^,#^	23.00 ± 2.00 *^,#^
	50 µg/mL	68.33 ± 1.53 *^,#^	0.33 ± 0.58 *^,#^	1.33 ± 0.58 *^,#^	30.00 ± 1.00 *^,#^
	100 µg/mL	72.33 ± 0.58 *^,#^	0.67 ± 0.58 ^#^	0.00 ± 0.00 *^,#^	27.00 ± 1.00 *^,#^
**Fe + orange BUE**	6.25 µg/mL	58.33 ± 0.58 *^,#^	1.67 ± 0.58	2.00 ± 2.00 *^,#^	38.00 ± 2.00 *
	12.5 µg/mL	66.33 ± 3.51 *^,#^	1.33 ± 0.58	3.00 ± 0.01 *^,#^	29.33 ± 3.51 *^,#^
	25 µg/mL	68.00 ± 3.61 *^,#^	1.33 ± 1.15	0.00 ± 0.00 *^,#^	30.67 ± 2.52 *^,#^
	50 µg/mL	69.33 ± 7.51 *^,#^	4.00 ± 2.00	2.67 ± 1.53 *^,#^	24.00 ± 4.00 *^,#^
	100 µg/mL	63.67 ± 10.50 *^,#^	0.00 ± 0.00 *^,#^	0.67 ± 1.15 *^,#^	35.67 ± 11.50
**Fe + orange BMAE**	6.25 µg/mL	55.67 ± 0.58 *^,#^	0.00 ± 0.00 *^,#^	3.67 ± 1.15 ^#^	40.67 ± 1.53
	12.5 µg/mL	49.67 ± 8.50	3.00 ± 1.00	6.67 ± 1.15 ^#^	40.67 ± 8.50
	25 µg/mL	43.67 ± 6.51	1.67 ± 0.58	8.67 ± 2.08	46.00 ± 4.00
	50 µg/mL	62.67 ± 2.52 *^,#^	2.67 ± 0.58	2.67 ± 2.08 ^#^	32.00 ± 1.00 *^,#^
	100 µg/mL	72.67 ± 0.58 *^,#^	1.67 ± 1.53	0.67 ± 1.15 *^,#^	25.00 ± 0.01

Percentages of normal and abnormal sperm were analyzed using the Kruskal–Wallis test, followed by the Mann–Whitney U test. Data were obtained from nine replicates (*n* = 3). * indicates significant differences from the control group at *p* < 0.05; ^#^ indicates significant differences from the Fe group at *p* < 0.05; ^##^ indicates significant differences between the Fe and the control groups at *p* < 0.05.

## Data Availability

The authors declare that the data supporting the findings of this study are available within the article.
